# Impact of growth curve and dietary energy-to-protein ratio of broiler breeders on offspring quality and performance

**DOI:** 10.1016/j.psj.2022.102071

**Published:** 2022-07-26

**Authors:** J. Heijmans, M. Duijster, W.J.J. Gerrits, B. Kemp, R.P. Kwakkel, H. van den Brand

**Affiliations:** ⁎ De Heus Animal Nutrition B.V., Ede, the Netherlands; † Animal Nutrition Group, Department of Animal Sciences, Wageningen University, Wageningen, the Netherlands; ‡ Adaptation Physiology Group, Department of Animal Sciences, Wageningen University, Wageningen, the Netherlands

**Keywords:** broiler breeder, feed strategy, maternal nutrition, offspring, broiler

## Abstract

The impact of growth curve (**GC**) and dietary energy-to-protein ratio of broiler breeder hens on chick quality and broiler performance was investigated. Pullets (n = 1,536) were randomly allotted to 24 pens and assigned to 1 of 8 treatments from hatch onwards, according to a 2 × 4 factorial arrangement with 2 GC (standard growth curve = **SGC** or elevated growth curve = **EGC**, +15%) and 4 diets, differing in energy-to-protein ratio (96%, 100%, 104%, and 108% AME_n_ diet). At 28 and 36 wk of age, 60 hatching eggs per maternal pen were selected for incubation and 768-day-old broilers were assigned to 32 pens according to maternal treatment.

Broilers from EGC breeders were 1.9 g heavier at hatch (*P* < 0.001) and 36 g heavier at slaughter (*P* = 0.001) than broilers from SGC breeders due to a 1.0 g/d higher growth rate (*P* = 0.003) and 1.5 g/d higher feed intake (*P* = 0.006) from hatch to 32 d of age. An increase in breeder dietary energy-to-protein ratio resulted in a linear decrease in embryonic mortality in the first 3 d of incubation (β = -0.2% per % AME_n_; *P* = 0.05). At hatch, broiler BW decreased with an increasing breeder dietary energy-to-protein ratio (β = -0.1 g per % AME_n_; *P* = 0.001), whereas at slaughter broiler BW increased with an increasing breeder dietary energy-to-protein ratio (β = 3.2 g per % AME_n_; *P* = 0.02). This was due to a linear increase in growth rate (β = 0.1 g/d per % AME_n_; *P* = 0.004) and feed intake (β = 0.1 g/d per % AME_n_; *P* = 0.02). Additionally, an increase in breeder dietary energy-to-protein ratio resulted in a linear decrease in body weight corrected feed conversion ratio (β = -0.002 per % AME_n_; *P* = 0.002). Overall, it can be concluded that a higher GC of breeders and an increase in breeder dietary energy-to-protein ratio enhances offspring performance.

## INTRODUCTION

A good day-old chick quality is crucial for health, welfare, and performance of broilers (Tona et al., 2005; Van de Ven et al., 2012). Most of the research on improving chick quality has focused on factors postoviposition and during incubation, for example, egg handling, egg storage, incubation temperature, and humidity (Molenaar et al., 2010b; Narinç and Aydemir, 2021). Recently, also potential effects of maternal nutrition on chick quality has gained more interest (Moraes et al., 2014, 2019; Lesuisse et al., 2017, 2018; Zukiwsky et al., 2021a).

Breeder dietary energy-to-protein ratio might be an important factor for day-old chick quality (Spratt and Leeson, 1987) and offspring performance (Moraes et al., 2014). So far, results have been inconsistent. Lesuisse et al. (2017, 2018) observed a 3.4 to 4 g lower day-old chick weight, but a 38 to 179 g higher BW at slaughter and a 0.03 lower feed conversion ratio in offspring from breeders that were fed 25% less dietary CP during rearing and production compared to breeders fed according to breeder recommendations. An 11 to 16% reduction in breeder dietary CP (compared to breeder recommendations), during the rearing phase alone did not affect day-old chick quality or offspring performance (Van Emous et al., 2015a; Moraes et al., 2019) or resulted in a 120 g lower BW at 36 d of age in female broilers (Moraes et al., 2014). In the studies of Moraes et al. (2014, 2019), however, dietary treatments were confounded with breeder BW, which has been shown to affect offspring performance as well (Bowling et al., 2018). These results may suggest that day-old chick quality and offspring performance benefit from a higher breeder dietary energy-to-protein ratio during both rearing and production. In all mentioned studies, the higher breeder dietary energy-to-protein ratio was realized by decreasing the CP level in the diet. It remains unclear whether or not a higher breeder dietary energy-to-protein ratio, by increasing dietary energy, during both the rearing and production phase affects chick quality and offspring performance.

Besides the maternal dietary energy-to-protein ratio, also severity of feed restriction might affect day-old chick quality and offspring performance. It has been observed that maternal feed restriction resulted in an increased risk of chronic metabolic diseases in offspring in mammals (Roseboom et al., 2006) and broilers (Van der Waaij et al., 2011). Broiler breeders are commonly fed restricted quantities of feed to control the growth trajectory and BW in order to ensure reproductive performance (Robinson et al., 1991; Bruggeman et al., 1999; Hocking et al., 2002; Sun et al., 2006). Recently, it is suggested that a higher growth curve, by means of an increased feed intake, is possible in modern broiler breeders without negative effects on egg production (Van der Klein et al., 2018; Heijmans et al., 2021; Zukiwsky et al., 2021b).

The aim of this study was to evaluate effects of growth curve and dietary energy-to-protein ratio of broiler breeder hens during rearing and production on day-old chick quality and offspring performance.

## MATERIALS AND METHODS

### Experimental Design

Female Ross 308 broiler breeder pullets (n = 1,536) were assigned to 1 of 8 treatments from hatch to 60 wk of age, according to a 2 × 4 factorial arrangement with 2 growth curves (**GC**) (standard growth curve = **SGC** or elevated growth curve = **EGC**) and 4 diets, differing in energy-to-protein ratio by step-wise increase in energy content from 96 to 108% AME_n_ at a similar CP content (further defined as 96%, 100%, 104%, and 108% AME_n_ diet), where the 100% AME_n_ treatment was the AME_n_ recommended by the breeding company (Aviagen, 2016a). The weekly growth target of the SGC was according to the breeder recommendation (Aviagen, 2016b), whereas the EGC targeted a 15% higher weekly growth relative to the SGC throughout rearing and production. Pair-gain of pullets within each GC was achieved by weekly adaptation of feed allocation per diet based on weekly BW measurement. Treatments were randomly assigned at the start of the experiment to 24 pens (64 pullets per pen) within 3 blocks (n = 3 pens per treatment). A detailed description of this experiment, including diet composition, was reported by Heijmans et al. (2021). At 28 and 36 wk of age, hatching eggs produced by these broiler breeders were incubated and broiler performance was recorded until slaughter. All experimental protocols were approved by the Central Commission on Animal Experimentation (The Hague, the Netherlands), approval number 2018.W-0023.001 and 2018.W-0023.002.

### Incubation

At 28 and 36 wk of age of the breeders, 60 clean settable hatching eggs per maternal pen (n = 1,440) were selected for incubation. Of each maternal pen, 20 hatching eggs of 3 consecutive days were selected based on the average egg weight per treatment ± 2.5 g. The eggs were stored at the breeder farm at 17°C for 10 to 12 d before incubation. Eggs were transported for approximately 1 h to the hatchery (Lagerwey, Lunteren, the Netherlands). Hatching eggs were incubated in a single-stage incubator with a maximum capacity of 4,800 hatching eggs (HatchTech, Veenendaal, the Netherlands). The incubator contained 1 trolley with 2 rows of 16 setter trays. Per maternal pen, the 60 selected hatching eggs were distributed evenly over 1 setter tray, resulting in 24 setter trays in total. The setter trays were randomly divided over 3 blocks in the incubator (top, middle, bottom of the trolley). Per row, the bottom 2 setter trays and the top 2 setter trays were kept empty. Eggs were warmed linearly in 10 h from storage temperature to an eggshell temperature (**EST**) of 37.8⁰C. The moment the eggs reached an EST of 37.8⁰C was considered as embryonic day (**E**)0 and the start of incubation. The EST was monitored throughout incubation, from start of the warming profile, using 4 sensors (NTC Thermistors: type DC 95; Thermometrics, Somerset, UK) that were attached to 4 individual eggs from different treatments. The EST sensors were attached to the eggshell at the equator of the egg, using a small piece of tape (Tesa BV, Almere, the Netherlands) in silicone heat sink compound paste (Type 340; Dow Corning, Midland, MI). The air temperature of the incubator was continuously adapted to maintain an EST of 37.8⁰C, based on the median temperature of the 4 EST sensors. At E8, all eggs were candled and clear eggs and eggs containing a dead embryo were removed. Eggs were turned over 90⁰ every hour until E18.

At E18, EST sensors were removed and all eggs were candled again and clear eggs or eggs containing a dead embryo were removed. Eggs containing viable embryos were transferred per setter tray to 1 hatching basket, resulting in 24 hatching baskets in total. The hatching baskets were placed on a trolley containing 3 rows of 11 hatching baskets. Per row, the bottom 2 hatching baskets and the top basket were kept empty. The hatching baskets were randomly divided over the top, bottom, middle, front, and back of the trolley. The trolley was placed in another incubator (HatchTech, Veenendaal, the Netherlands), where 6 EST sensors were attached to 6 individual eggs from different treatments as described above. Again, the air temperature of the incubator was continuously adapted to maintain an EST of 37.8⁰C based on the median temperature of the 6 EST sensors. From 467 h after the start of incubation (E19h11), the EST sensors were removed and the air temperature of the incubator was fixed at the current settings until pull of the hatched chicks (E21h13). Relative humidity was maintained between 50 and 65% until E4, between 50 and 60% from E4 to E7, between 50 and 55% from E7 to E10, and between 40 and 45% thereafter. Carbon dioxide was maintained below 0.35% throughout incubation.

### Hatching

From E19h11 until pull (E21h13), every 6 h the incubator was opened to check whether or not chicks had hatched. All chicks that hatched were marked with a permanent marker on the head. Six hours later, marked chicks were collected and chick quality was scored as described below. After assessing chick quality, first grade chicks were transferred to another similar incubator (HatchTech, Veenendaal, the Netherlands), where they were placed in 24 hatching baskets until pull. After pulling, all chicks were feather sexed and pooled per maternal treatment and sex.

### Broilers, Housing, and Management

At each maternal age (28 and 36 wk of age), 384 female and 384 male first-grade chicks were transported for 1 h to the broiler facility (Eerde, the Netherlands) in a climate controlled truck. At the start of the experiment (d 0), the maternal treatments were randomly assigned to 64 floor pens within 4 blocks (n = 8 pens per treatment) in a climate controlled room. In each pen, 6 female and 6 male broilers were placed, originating from the same maternal treatment. Broilers were marked with a unique neck tag number. At 7 d of age, 1 female and 1 male broiler were removed per pen, euthanized by cervical dislocation and stored until further analysis. At 14 d of age, 2 adjacent pens of the same maternal treatment were merged (n = 4 pens per treatment). Each pen (1 m^2^ from 0 to 14 d of age and 2 m^2^ from 14 d of age onwards) contained wood shavings as bedding. Water and feed were supplied ad libitum via drinking nipples and a feeding trough, respectively. At d 0, photoperiod was 23L:1D (40 lux), which gradually changed to 18L:6D at d 3, which was maintained until slaughter. Temperature was set at 33°C at d 0 and decreased gradually to 21°C at d 32. Broilers were fed a standard commercial available broiler diet according to a 4-phase feeding program. A starter diet (2,925 kcal of AME_n_/kg, 198 g/kg CP, and 11.6 g digestible lysine/kg) from d 0 to 7, a grower I diet (3,000 kcal of AME_n_/kg, 187 g/kg CP and 10.7 g digestible lysine/kg) from d 7 to 21, a grower II diet (3,050 kcal of AME_n_/kg, 180 g/kg CP and 10.0 g digestible lysine/kg) from d 21 to 28 and a finisher diet (3,100 kcal of AME_n_/kg, 180 g/kg CP and 9.8 g digestible lysine/kg) from d 28 to 32.

### Measurements

#### Egg Weight

Selected hatching eggs were weighed individually before storage, at start of incubation (E0) and at E18. Egg weight (**EW**) loss during storage was calculated as the difference between EW before storage and E0. Egg weight loss during incubation was calculated as the difference between EW at E0 and EW at E18.

#### Fertility, Hatchability, Embryonic Mortality

Clear eggs and eggs containing a dead embryo at E8 and E18 and unhatched eggs at pull were opened to determine infertility or stage of embryonic mortality. The following classifications were used: 1) unfertilized eggs showing no signs of development, 2) very early mortality (E0-E3): area vasculosa until start development black eye (<0.5 mm), 3) early embryonic mortality (E4-E10); black eye (>0.5 mm) until feather development, 4) mid embryonic mortality (E11-E18): small embryo with feathers, 5) late embryonic mortality (E19-E21.5): full grown embryo. Embryos showing clear deformities were noted as abnormal embryos. Fertility was calculated as a percentage of set eggs. Hatchability was calculated as a percentage of set eggs and of fertile eggs. Embryonic mortality was calculated as a percentage of fertile eggs.

#### Chick Quality and Hatch Window

From E19h11, every 6 h chick quality of just hatched chicks was determined. Chicks were classified as first or second grade. A chick was classified as first grade when it was dry, clean, and free of deformities and with bright eyes. The other chicks were classified as second grade, including the chickens that died in the hatching basket after emergence from the egg shell. Second grade chicks were euthanized by cervical dislocation. Percentage first and second grade chicks were calculated relative to the total number of hatched chicks. Body weight of all chicks was determined. Hereafter, first grade chicks were scored on activity and navel, beak, and leg quality. Activity was scored as good or weak, after placing the chick on its back. If the chick returned to the standing position within 2 s, it was noted as good; longer than 2 s was noted as weak. Navel quality was scored as 0 (closed and clean navel), 1 (black button up to 2 mm or black string), or 2 (black button exceeding 2 mm) (Molenaar et al., 2010a). Beak quality was scored as 0 (normal beak), or 1 (red dot or nostrils contaminated with albumen). Leg quality was scored as 0 (normal legs, toes and hocks), 1 (red or swollen hock of 1 leg) or 2 (red or swollen hocks from both legs). Every fifth first grade chick was euthanized by cervical dislocation, followed by decapitation and the residual yolk (**RY**) was removed and weighed. In total, 10 chicks per setter tray were euthanized for determination of RY weight. Yolk-free body mass (**YFBM**) was calculated as chick weight minus RY weight. Start of hatch was determined per setter tray as the time of hatch of the first chick. The hatch window was calculated per setter tray as the time of hatch of the last chick minus the time of hatch of the first chick.

#### Broiler Performance

Broilers were weighed individually at d 0, 4, 7, 14, 21, 28 and 32 and feed intake was determined per pen on those weighing days. Average daily gain (**ADG**), average daily feed intake (**ADFI**) and feed conversion ratio (**FCR)** were calculated between those days. Feed conversion ratio over the whole period (d 0–32) was corrected for differences in BW at day 32 (**FCRc**). Heavier birds are assumed to have a higher maintenance requirement and a higher feed intake. Therefore, the equation is based on the assumption that 0.03 FCR is equivalent to a 100 g difference in BW (Van Krimpen et al., 2019). A standard BW of 2100 g was used for calculation of FCRc:

$$FCRc=FCR+\frac{2100-actual\;BW\;d\;32}{100}*0.03$$

#### Processing Yields and Myopathies

At d 32, 2 male and 2 female broilers per pen were randomly selected and weighed. Hereafter, these broilers were euthanized by a percussive blow to the head, followed by cervical dislocation. The head, skin, legs, tips of the wing (*manus*), tail, and visceral organs were removed, leaving the wet carcass to be weighed. The pectoralis major, pectoralis minor, thighs plus drums, and wings were removed and weighed separately. Slaughter yield of each of these components was calculated as a percentage of the wet carcass. The pectoralis major was scored on appearance of wooden breast and white striping (adopted from Kuttappan et al., 2016). White striping was scored as 0 (no white striations), 1 (small thin white striations <1 mm) or 2 (thick white striations 1–2 mm). Wooden breast was scored as 0 (soft breast muscle), 1 (part of the breast muscle is hardened), or 2 (whole breast muscle is hardened). Prevalence of white striping or wooden breast was calculated as percentage of broilers with a score 1 or 2 of the total broilers slaughtered.

### Statistical Analysis

All continuous and binomial data were analyzed, using the Restricted Maximum Likelihood variance component analysis procedure within a generalized linear mixed model (Genstat 19th Edition, 2019). Means and model residuals were checked on homogeneity of variance prior to analyses. Not-normal distributed data (early embryonic mortality and abnormal embryos) were log transformed before analyses. None of the models included the interaction of GC or diet with breeder age, as this was confounded with season and incubator. For statistical analysis of incubation parameters, the experimental unit was setter tray. The model used for incubation parameters was:

(1) $$Y_{ijk}=\;\mu +GC_{i}+Diet_{j}+GC_{i}\;x\;Diet_{j}+Age_{k}+e_{ijk}$$

Where Y_ijk_ is the dependent variable, µ is the overall mean, GC_i_ is the growth curve of the breeders (i = SGC or EGC), Diet_j_ is the energy-to-protein ratio in the diet of the breeders (j = 96%, 100%, 104%, or 108% AME_n_), GC_i_ x Diet_j_ is the interaction between growth curve and diet, Age_k_ is age of the breeder flock (k = 28 or 36 wk of age), and e_ijk_ is the residual error. Block in the incubator was added to the model as a random factor. For ADG, ADFI, FCR and FCRc model 1 was also used, without Block. Pen was considered as the experimental unit.

For analysis of chick quality at hatch, model 1 was used, added with sex and its interactions with Diet and GC:

(2) $$Y_{ijkl}=\;\mu +GC_{i}+Diet_{j}+GC_{i}\;x\;Diet_{j}+Age_{k}+Sex_{l}+GC_{i}\;x\;Sex_{l}+Diet_{j}x\;Sex_{l}+\;GC_{i}\;x\;Diet_{j}x\;Sex_{l}+e_{ijkl}$$

where Y_ijkl_ is the dependent variable, µ is the overall mean, GC_i_ is the growth curve of the breeders (i = SGC or EGC), Diet_j_ is the energy-to-protein ratio in the diet of the breeders (j = 96%, 100%, 104% or 108% AME_n_), GC_i_ x Diet_j_ is the interaction between growth curve and diet, Age_k_ is age of the breeder flock (k = 28 or 36 wk of age), Sex_l_ is the sex of the chick (l = male of female), GC_i_ x Sex_l_ is the interaction between growth curve and sex, Diet_j_ x Sex_l_ is the interaction between diet and sex, GC_i_ x Diet_j_ x Sex_l_ is the interaction between growth curve, diet and sex, and e_ijkl_ is the residual error. Hatching tray was considered as the experimental unit and was added to the model as a random factor.

For broiler BW data and slaughter characteristics model 2 was used, with pen (n = 16 per treatment up to d 14; n = 8 per treatment after d 14) added to the model as a random factor instead of hatching tray. Pen was considered as the experimental unit. Preliminary analysis showed that interactions between GC and Sex, Diet and Sex, and between GC, Diet and Sex were not significant for any of the variables. Furthermore, preliminary analysis showed that inclusion of Sex in the model did not affect results of the other factors. Consequently, the factor Sex, the interaction with Sex and the random factor were excluded from the model.

Fisher adjustments were used for multiple comparisons of factorial analysis. Additionally, linear and quadratic contrasts of Diet and Diet x GC interaction were analyzed. If linear effects of dietary energy-to-protein ratio were observed, also within GC, the slope (β) is presented. If quadratic effects of dietary energy-to-protein ratio, also within GC, were observed, the estimated AME_n_ percentage at which the dependent variable was at the maximum (concave quadratic relation) or minimum (convex quadratic relation) was calculated and presented. Data are presented as LSmeans ± SEM. For transformed data, LSmeans of original data are presented, combined with *P*-values of the transformed data. Differences were reported where *P* ≤ 0.05.

## RESULTS

### Incubation

No interaction between breeder GC and dietary energy-to-protein ratio was observed on hatching EW and EW loss during storage and incubation (Table 1). Hatching eggs obtained from EGC breeders were 2.4 g heavier before storage, 2.4 g heavier at E0, and 2.3 g heavier at E18 than hatching eggs from SGC breeders (*P* < 0.001; Table 1). A negative linear effect of an increasing breeder dietary energy-to-protein ratio was observed on hatching EW before storage (β = -0.06 g per % AME_n_), at E0 (β = -0.06 g per % AME_n_), and at E18 (β = -0.04 g per % AME_n_; *P* ≤ 0.03; Table 1). No differences were observed between breeder GC or dietary energy-to-protein ratio on egg weight loss during storage and incubation (Table 1).

**Table 1:** Average egg weight (EW) and EW loss during storage and incubation of hatching eggs obtained from broiler breeders at 2 different ages (28 and 36 wk of age), which were fed to reach one of two targeted growth curves (SGC = standard growth curve or EGC = elevated growth curve [+15%]) and 4 diets, differing in energy-to-protein ratio (96, 100, 104, or 108% AME_n_) from hatch onwards.

		EW (g)^1^			EW loss %^1^	
Item		Before storage^2^	E0^3^	E18^3^	During storage	During incubation
Growth curve (n = 24)						
SGC		58.1^b^	57.6^b^	52.3^b^	0.89	9.09
EGC		60.5^a^	60.0^a^	54.6^a^	0.87	8.98
SEM		0.1	0.1	0.1	0.03	0.05
Diet (n = 12)						
96% AME_n_		59.6^a^	59.1^a^	53.6	0.92	9.15
100% AME_n_		59.6^a^	59.1^a^	53.7	0.85	9.08
104% AME_n_		59.0^b^	58.5^b^	53.2	0.89	8.94
108% AME_n_		59.0^b^	58.5^b^	53.2	0.86	8.97
SEM		0.2	0.2	0.1	0.03	0.07
Treatment (n = 6)						
SGC	96% AME_n_	58.4	57.8	52.4	0.95	9.23
	100% AME_n_	58.1	57.7	52.4	0.80	9.18
	104% AME_n_	58.0	57.4	52.2	0.90	9.00
	108% AME_n_	58.0	57.4	52.3	0.89	8.96
EGC	96% AME_n_	60.9	60.4	54.9	0.90	9.07
	100% AME_n_	61.0	60.5	55.0	0.90	9.00
	104% AME_n_	60.1	59.6	54.3	0.87	8.87
	108% AME_n_	60.1	59.6	54.1	0.83	8.97
	SEM	0.2	0.2	0.2	0.05	0.10
Hen age (n = 24)						
28 wk		55.6^b^	55.1^b^	50.2^b^	0.94^a^	8.74^b^
36 wk		63.1^a^	62.5^a^	56.7^a^	0.82^b^	9.33^a^
SEM		0.12	0.11	0.04	0.03	0.04
*P*-value						
Growth curve (GC)		<0.001	<0.001	<0.001	0.68	0.10
Diet (factorial)		0.003	0.004	0.08	0.31	0.12
Diet (linear)		<0.001	0.001	0.03	0.28	0.23
Diet (quadratic)		0.89	0.98	0.84	0.42	0.37
GC x Diet (factorial)		0.16	0.24	0.25	0.22	0.73
GC x Diet (linear)		0.12	0.130	0.09	0.59	0.55
GC x Diet (quadratic)		0.55	0.66	0.53	0.16	0.12
Hen age		<0.001	<0.001	<0.001	<0.001	0.001

ab LSmeans within a column and factor lacking a common superscript differ (*P* ≤ 0.05).

1 EW = egg weight; EW loss during storage (10–12 d) = (EW before storage–EW E0)/(EW before storage)*100%; EW loss during incubation = (EW E0–EW E18)/(EW E0)*100%.

2 Per replicate 60 hatching eggs of 3 consecutive days (20 hatching eggs per day) were selected and stored at 17°C for 10 to 12 d before incubation.

3 Embryonic day (E).

No interaction between breeder GC and dietary energy-to-protein ratio was observed on fertility, hatchability, very early (E0-E3), early (E4-E10) or mid (E11-E18) embryonic mortality, start of hatch, hatch window or percentage of second grade chicks (Table 2). Increasing dietary energy-to-protein ratio linearly reduced embryonic mortality within embryos from SGC breeders (β = -0.3% per% AME_n_,), but not in embryos from EGC breeders (β = 0.1% per% AME_n_; *P* = 0.03; Table 2). This occurred mainly during the last 3 d of incubation (*P* = 0.05; Table 2). Very early (E0-E3) embryonic mortality was not affected by breeder GC, but there was a linear effect of breeder dietary energy-to-protein ratio. An increase in breeder dietary energy-to-protein ratio resulted in a linear decrease in very early embryonic mortality (β = -0.2% per% AME_n_; *P* = 0.05; Table 2). No effect of breeder GC or dietary energy-to-protein ratio was observed on fertility, hatchability, early or mid-embryonic mortality, start of hatch, hatch window, or percentage of second grade chicks (Table 2).

**Table 2:** Fertility, hatchability, embryonic mortality, start of hatch, hatch window and percentage second grade chicks obtained from broiler breeders at 2 different ages (28 and 36 wk of age), which were fed to reach one of two targeted growth curves (SGC = standard growth curve or EGC = elevated growth curve [+15%]) and 4 diets, differing in energy-to-protein ratio (96, 100, 104, or 108% AME_n_) from hatch onwards.

					Embryonic mortality (% of fertile eggs)								
Item		Fertility of set eggs (%)	Hatch. of set eggs (%)	Hatch. of fertile eggs (%)	E0-E3^1^	E4-E10	E11-E17	E18-E21.5	E0-E21.5	Abnormal	Start hatch (h)	Hatch window (h)	Second grade chicks (% of total chicks)
Growth curve (n = 24)													
SGC		98.7	91.9	93.1	3.5	0.8	0.3	1.4	6.1	0.8	485.5	24.8	0.8
EGC		97.9	91.6	93.6	3.1	0.8	0.5	1.7	6.0	0.4	485.5	26.3	0.6
SEM		0.4	0.8	0.6	0.5	0.3	0.1	0.4	0.6	0.2	0.7	0.8	0.2
Diet (n = 12)													
96% AME_n_		98.2	91.5	93.2	4.1	0.7	0.3	1.3	6.4	0.4	486.5	24.0	0.3
100% AME_n_		98.7	90.9	92.3	3.7	0.9	0.3	1.8	6.8	0.9	485.0	24.5	0.5
104% AME_n_		98.2	91.8	93.5	3.1	0.8	0.3	2.0	6.2	0.3	485.0	27.0	1.1
108% AME_n_		98.2	92.8	94.5	2.3	0.6	0.7	1.1	4.7	0.8	485.5	26.5	0.9
SEM		0.6	1.1	0.9	0.7	0.4	0.2	0.5	0.8	0.3	1.0	1.2	0.3
Treatment (n = 6)													
SGC	96% AME_n_	98.1	90.3	92.1	4.5	0.9	0.3	1.7	7.4	0.6	486.0	23.0	0.3
	100% AME_n_	99.4	90.8	91.3	4.5	0.8	0.3	2.0	7.6	1.1	484.0	25.0	0.6
	104% AME_n_	98.1	91.9	93.8	3.1	1.1	0.3	1.4	5.9	0.3	486.0	27.0	1.5
	108% AME_n_	99.2	94.4	95.2	2.0	0.3	0.6	0.6	3.3	1.4	486.0	24.0	0.9
EGC	96% AME_n_	98.3	92.7	94.3	3.7	0.6	0.3	0.9	5.4	0.3	487.0	25.0	0.3
	100% AME_n_	97.9	91.1	93.3	2.9	1.1	0.4	1.6	6.0	0.6	486.0	24.0	0.5
	104% AME_n_	98.3	91.6	93.1	3.1	0.6	0.3	2.6	6.5	0.3	484.0	27.0	0.6
	108% AME_n_	97.2	91.1	93.7	2.6	0.9	0.9	1.7	6.0	0.3	485.0	29.0	0.9
	SEM	0.8	1.6	1.3	1.0	0.6	0.3	0.7	1.2	0.4	1.3	1.7	0.5
Hen age (n = 24)													
28 wk		98.7	91.4	92.6	3.9	0.7	0.2	2.0	6.9	0.5	485.8	25.3	0.8
36 wk		97.9	92.1	94.1	2.6	0.8	0.6	1.0	5.1	0.7	485.2	25.8	0.6
SEM		0.4	0.7	0.6	0.5	0.3	0.1	0.5	0.6	0.1	0.8	0.7	0.2
*P*-value													
Growth curve (GC)		0.23	0.81	0.60	0.59	0.87	0.68	0.50	1.00	0.20	1.00	0.22	0.37
Diet (factorial)		0.86	0.65	0.30	0.26	0.87	0.45	0.41	0.23	0.54	0.66	0.24	0.27
Diet (linear)		0.83	0.32	0.17	0.05	0.54	0.21	0.90	0.10	0.83	0.49	0.07	0.09
Diet (quadratic)		0.63	0.44	0.23	0.68	0.60	0.34	0.10	0.19	0.95	0.30	0.68	0.50
GC x Diet (factorial)		0.38	0.29	0.31	0.74	0.77	0.96	0.23	0.16	0.79	0.45	0.32	0.70
GC x Diet (linear)		0.34	0.06	0.07	0.34	0.54	0.66	0.05	0.03	0.67	0.25	0.36	0.82
GC x Diet (quadratic)		0.82	0.85	0.88	0.67	0.94	0.76	0.74	0.63	0.54	1.00	0.10	0.35
Hen age		0.23	0.52	0.15	0.13	0.51	0.11	0.23	0.09	0.43	0.67	0.66	0.67

1 Embryonic day (E).

### Chick Quality

In total 2,598 first grade chicks hatched and were scored on chick quality, of which 480 chicks were dissected for RY and YFBM weight. No interaction between breeder GC and dietary energy-to-protein ratio was observed on hatchling weight, RY weight, YFBM, activity or beak score (Table 3). Increasing dietary energy-to-protein ratio linearly increased percentage of chicks with navel score 1 within chicks from SGC breeders (β = 0.4% per % AME_n_,), but not in chicks from EGC breeders (β = -0.5% per % AME_n_; *P* = 0.03; Table 3). A quadratic interaction between breeder GC and dietary energy-to-protein ratio on percentage of chicks with leg score 1 was observed (*P* = 0.04; Table 3). The lowest percentage of chicks with leg score 1 was estimated at 103% AME_n_ (∆_max_ = -8.8%) for chicks obtained from SGC breeders, whereas this was estimated at 103% AME_n_ (∆_max_ = 8.0%) for chicks from EGC breeders. Percentage of chicks with leg score 2 did not differ between treatments (Table 3).

**Table 3:** Hatchling weight (HW), residual yolk (RY) weight, yolk-free body mass (YFBM), activity, navel, beak and leg score of chicks obtained from broiler breeders at 2 different ages (28 and 36 wk of age), which where were fed to reach one of two targeted growth curves (SGC = standard growth curve or EGC = elevated growth curve [+15%]) and 4 diets, differing in energy-to-protein ratio (96, 100, 104, or 108% AME_n_), from hatch onwards.

						Navel score^2^ (%)			Leg score^4^ (%)	
Item		HW (g)	RY (g)	YFBM (g)	Activity^1^ (%)	Score 1	Score 2	Beak^3^ (%)	Score 1	Score 2
Growth curve (n = 24)										
SGC		41.9^b^	5.1^b^	36.9^b^	11.1	43.8	12.5^b^	11.0	23.7	8.2
EGC		43.8^a^	5.7^a^	38.1^a^	12.5	44.0	16.2^a^	9.6	21.9	8.4
SEM		0.1	0.1	0.1	0.9	0.9	1.4	1.1	1.5	1.1
Diet (n = 12)										
96% AME_n_		43.1^a^	5.3	37.5	13.0	46.0	14.2	11.1	23.3	8.6
100% AME_n_		43.1^a^	5.5	37.8	11.8	41.2	15.1	10.2	20.7	7.4
104% AME_n_		42.7^b^	5.4	37.3	12.3	44.1	13.1	9.6	25.1	8.2
108% AME_n_		42.6^b^	5.3	37.3	10.1	44.1	15.2	10.3	22.1	8.9
SEM		0.1	0.1	0.2	1.5	1.5	1.8	1.4	2.1	1.5
Treatment (n = 6)										
SGC	96% AME_n_	42.1	5.1	37.0	13.1	41.6^b^	13.6	10.6	26.3	7.9
	100% AME_n_	42.0	5.2	36.9	8.9	42.9^b^	11.8	11.1	17.5	8.6
	104% AME_n_	41.8	5.2	36.9	13.9	44.6^ab^	10.1	8.9	22.0	5.9
	108% AME_n_	41.8	5.0	36.6	8.4	45.9^ab^	14.6	13.2	21.8	10.2
EGC	96% AME_n_	44.2	5.6	38.1	12.8	50.5^a^	14.7	11.6	20.3	9.3
	100% AME_n_	44.2	5.7	38.7	14.7	39.5^b^	18.4	9.3	23.8	6.3
	104% AME_n_	43.5	5.7	37.6	10.8	43.5^b^	16.1	10.2	28.3	10.6
	108% AME_n_	43.4	5.6	38.1	11.7	42.3^b^	15.8	7.3	22.5	7.7
	SEM	0.2	0.2	0.2	2.2	2.2	2.5	2.0	2.9	2.1
Hen age (n = 24)										
28 wk		40.0^b^	4.8^b^	35.1^b^	10.6	48.9^a^	9.5^b^	7.1^b^	15.4^b^	7.8
36 wk		45.7^a^	6.0^a^	39.9^a^	12.9	38.8^b^	19.3^a^	13.4^a^	30.2^a^	8.8
SEM		0.1	0.1	0.1	0.6	0.5	1.5	1.1	1.4	1.1
*P*-value										
Growth curve (GC)		<0.001	<0.001	<0.001	0.38	0.91	0.04	0.33	0.39	0.85
Diet (factorial)		0.007	0.75	0.11	0.61	0.24	0.82	0.89	0.50	0.90
Diet (linear)		0.001	0.83	0.17	0.28	0.71	0.89	0.62	0.92	0.79
Diet (quadratic)		0.76	0.27	0.62	0.75	0.16	0.74	0.56	0.93	0.54
GC x Diet (factorial)		0.29	0.99	0.13	0.23	0.03	0.53	0.25	0.14	0.26
GC x Diet (linear)		0.12	0.83	0.77	0.92	0.03	0.98	0.16	0.30	0.73
GC x Diet (quadratic)		0.53	0.79	0.86	0.97	0.15	0.14	0.45	0.04	0.57
Hen age		<0.001	0.004	<0.001	0.07	<0.001	0.009	0.02	0.002	0.56

ab LSmeans within a column and factor lacking a common superscript differ (*P* ≤ 0.05).

1 Percentage of chicks scored as weak.

2 Percentage of chicks with a score 1 (black button up to 2 mm or black string) or a score 2 (black button exceeding 2 mm or open navel.

3 Percentage of chicks with a red dot on beak or nostrils contaminated with albumen.

4 Percentage of chicks with a score 1 (red or swollen hock of 1 leg) or a score 2 (red or swollen hocks from both legs).

Hatchlings obtained from EGC breeders where 1.9 g heavier, with a 0.6 g heavier RY and 1.2 g heavier YFBM, compared to hatchlings from SGC breeders (*P* < 0.001; Table 3). An increase in breeder dietary energy-to-protein ratio resulted in a linear decrease in hatchling weight (β = -0.1 g per % AME_n_; *P* = 0.001), but did not affect RY weight or YFBM (Table 3). Percentage of chicks with navel score 2 was 3.8% higher in chicks from EGC breeders, compared to SGC breeders (*P* = 0.04; Table 3). Breeder dietary energy-to-protein ratio did not affect chick navel quality.

### Broiler Performance

A linear interaction between breeder GC and dietary energy-to-protein ratio was observed on broiler BW at 0, 4, 7, 14, and 21 d of age (*P* < 0.05; Table 4). At 0 and 4 d of age, broiler BW decreased with an increasing breeder dietary energy-to-protein ratio, but this was more profound in broilers from EGC breeders (β = -0.19 g per % AME_n_ on average) than from SGC breeders (β = -0.03 g per % AME_n_ on average). At 7, 14 and 21 d of age, broiler BW increased linearly with an increasing dietary energy-to-protein ratio within broilers from SGC breeders (β = 1.2 g per % AME_n_ on average), whereas this was not observed within broilers from EGC breeders (β = -0.3 g per % AME_n_ on average). At 28 and 32 d of age, this interaction was not observed anymore, but broilers obtained from EGC breeders were 33 g (*P* < 0.001) and 36 g (*P* = 0.001) heavier, respectively, than broilers from SGC breeders (Table 4). Furthermore, at 28 and 32 d of age, an increase in breeder dietary energy-to-protein ratio resulted in a linear increase in broiler BW (β = 2.3 g per % AME_n_ and β = 3.2 g per % AME_n_; *P* = 0.02 and *P* = 0.007, respectively).

**Table 4:** BW at different ages of broilers obtained from broiler breeders at 2 different ages (28 and 36 wk of age), which were fed to reach one of two targeted growth curves (SGC = standard growth curve or EGC = elevated growth curve [+15%]) and 4 diets, differing in energy-to-protein ratio (96, 100, 104, or 108% AME_n_), from hatch onwards.

		BW^1^ (g)						
Item		0 d	4 d	7 d	14 d	21 d	28 d	32 d
Growth curve								
SGC		39.3	96.8^b^	158.2	453.9^b^	935.9^b^	1,632^b^	2,090^b^
EGC		41.7	101.7^a^	164.9	468.1^a^	957.9^a^	1,665^a^	2,126^a^
SEM		0.1	0.3	0.5	1.7	3.4	6	7
Diet								
96% AME_n_		40.9	100.3^a^	163.2	458.7^b^	934.0^b^	1,627^b^	2,077^b^
100% AME_n_		40.7	99.1^ab^	160.8	458.1^b^	947.8^a^	1,650^a^	2,115^a^
104% AME_n_		40.2	99.3^ab^	161.4	467.6^a^	959.3^a^	1,666^a^	2,124^a^
108% AME_n_		40.2	98.3^b^	160.7	459.8^b^	946.4^ab^	1,652^a^	2,117^a^
SEM		0.1	0.4	0.7	2.3	4.8	8	11
Treatment								
SGC	96% AME_n_	39.6^c^	96.9	158.7^c^	449.3	916.9	1,603	2,052
	100% AME_n_	39.3^cd^	96.7	156.9^c^	447.4	930.2	1,628	2,089
	104% AME_n_	39.1^d^	97.1	158.1^c^	462.1	952.8	1,656	2,112
	108% AME_n_	39.3^cd^	96.5	159.1^c^	456.8	943.5	1,642	2,108
EGC	96% AME_n_	42.2^a^	103.7	167.8^a^	468.1	951.1	1,650	2,101
	100% AME_n_	42.0^a^	101.5	164.7^b^	468.6	965.4	1,672	2,141
	104% AME_n_	41.3^b^	101.5	164.6^b^	473.2	965.8	1,677	2,135
	108% AME_n_	41.1^b^	100.1	162.4^b^	462.7	949.3	1,662	2,126
	SEM	0.2	0.6	1.0	3.3	6.8	12	15
Hen age								
28 wk		37.8^b^	97.4^b^	156.9^b^	450.1^b^	923.6^b^	1,618^b^	2,074^b^
36 wk		43.2^a^	101.1^a^	166.2^a^	472.0^a^	970.1^a^	1,679^a^	2,142^a^
SEM		0.1	0.3	0.5	1.7	3.4	6	7
*P*-value								
Growth curve (GC)		<0.001	<0.001	<0.001	<0.001	<0.001	<0.001	0.001
Diet (factorial)		<0.001	0.02	0.06	0.02	0.006	0.02	0.02
Diet (linear)		<0.001	0.003	0.03	0.24	0.03	0.02	0.007
Diet (quadratic)		0.36	0.83	0.23	0.14	0.008	0.03	0.04
GC x Diet (factorial)		0.02	0.08	0.04	0.09	0.08	0.51	0.58
GC x Diet (linear)		0.004	0.02	0.005	0.03	0.02	0.16	0.20
GC x Diet (quadratic)		0.23	0.57	0.50	0.44	0.67	0.98	0.83
Hen age		<0.001	<0.001	<0.001	<0.001	<0.001	<0.001	<0.001

a-d LSmeans within a column and factor lacking a common superscript differ (*P* ≤ 0.05).

1 At 14 d of age, 2 adjacent pens from the same treatment were merged. n = 16 per treatment for d 0, 4, 7, and 14, and n = 8 per treatment for d 21, 28, and 32.

Weekly broiler ADG, ADFI, and FCR can be found in supplementary Table S1. No interaction was observed on ADG, ADFI, or FCR over the whole period (0–32 d of age; Table 5). Broilers originating from EGC breeders had a 1.0 g/d higher ADG and 1.5 g/d higher ADFI over the whole period, compared to broilers originating from SGC breeders (*P* ≤ 0.006; Table 5). This was mainly due to a higher ADG and ADFI observed in the first 21 d of age (Supplementary Table S1). FCR did not differ between broilers from EGC and SGC breeders.

**Table 5:** Average daily gain (ADG; g/d), average daily feed intake (ADFI; g/d) and feed conversion ratios (FCR; kg of feed/kg of BW gain) of broilers from 0 to 32 d of age, obtained from broiler breeders at 2 different ages (28 and 36 wk of age), which were fed to reach one of two targeted growth curves (SGC = standard growth curve or EGC = elevated growth curve [+15%]) and 4 diets, differing in energy-to-protein ratio (96, 100, 104, or 108% AME_n_), from hatch onwards.

		d 0–32			
Item		ADG	ADFI	FCR	FCR_c_^1^
Growth curve (n = 32)					
SGC		64.1^b^	89.5^b^	1.40	1.40
EGC		65.1^a^	91.0^a^	1.40	1.39
SEM		0.2	0.4	0.01	0.01
Diet (n = 16)					
96% AME_n_		63.6^b^	89.0^b^	1.40	1.42^a^
100% AME_n_		64.9^a^	90.3^ab^	1.39	1.39^b^
104% AME_n_		65.1^a^	91.0^a^	1.40	1.39^b^
108% AME_n_		64.9^a^	90.7^a^	1.39	1.39^b^
SEM		0.3	0.5	0.01	0.01
Treatment (n = 8)					
SGC	96% AME_n_	62.9	87.9	1.40	1.43
	100% AME_n_	64.1	89.2	1.40	1.40
	104% AME_n_	64.8	90.6	1.40	1.39
	108% AME_n_	64.7	90.4	1.39	1.39
EGC	96% AME_n_	64.3	90.2	1.40	1.41
	100% AME_n_	65.6	91.5	1.39	1.38
	104% AME_n_	65.4	91.3	1.41	1.40
	108% AME_n_	65.2	90.9	1.40	1.38
	SEM	0.5	0.7	0.01	0.01
Hen age (n = 32)					
28 wk		63.7^b^	87.6^b^	1.36^b^	1.37^b^
36 wk		65.6^a^	92.9^a^	1.43^a^	1.42^a^
SEM		0.2	0.4	0.01	0.01
*P*-value					
Growth curve (GC)		0.003	0.006	0.33	0.18
Diet (factorial)		0.006	0.05	0.14	0.008
Diet (linear)		0.004	0.02	0.21	0.002
Diet (quadratic)		0.03	0.12	0.78	0.28
GC x Diet (factorial)		0.55	0.41	0.62	0.48
GC x Diet (linear)		0.19	0.12	0.62	0.25
GC x Diet (quadratic)		0.84	0.98	0.94	0.53
Hen age		<0.001	<0.001	<0.001	<0.001

ab LSmeans within a column and factor lacking a common superscript differ (*P* ≤ 0.05).

1 Corrected FCR to a standard BW of 2100 g, calculated as FCR – (2100 – actual BW d 32)/100*0.03.

An increase in breeder dietary energy-to-protein ratio resulted in a linear increase in ADG over the whole period (β = 0.1 g/d per % AME_n_; *P* = 0.004; Table 5). This was mainly due to a linear increase in ADG from 7 to 14 d of age (β = 4.4 g/d per % AME_n_; *P* = 0.03) and a quadratic relation with the highest ADG estimated at 103% AME_n_ (∆_max_ = 2.2 g/d; *P* = 0.04) from 14 to 21 d of age (Supplementary Table S1). An increase in breeder dietary energy-to-protein ratio resulted in a linear increase in ADFI over the whole period (β = 0.1 g/d per % AME_n_; *P* = 0.02; Table 5). This was mainly due to a linear increase in ADFI from 14 to 21 d of age (β = 0.2 g/d per % AME_n;_ *P* = 0.04) and from 21 to 28 d of age (β = 0.3 g/d per % AME_n_; *P* = 0.02; Supplementary Table S1). FCR did not differ between dietary energy-to-protein ratios. FCRc, however, decreased linearly with an increasing dietary energy-to-protein ratio (β = -0.002 per % AME_n_; *P* = 0.002; Table 5).

### Slaughter Characteristics

A linear interaction between breeder GC and dietary energy-to-protein ratio was observed on carcass yield percentage (*P* = 0.02) and thighs plus drums as percentage of the carcass (*P* < 0.001; Table 6). Carcass yield percentage increased linearly with an increasing breeder dietary energy-to-protein ratio in broilers from SGC breeders (β = 0.08% per % AME_n_), whereas it decreased linearly in broilers from EGC breeders (β = -0.09% per % AME_n_). Thighs plus drums as percentage of the carcass decreased linearly with an increasing breeder dietary energy-to-protein ratio in broilers from SGC breeders (β = -0.06% per % AME_n_), whereas it increased linearly in broilers from EGC breeders (β = 0.08% per % AME_n_). No effect of treatments was observed on pectoralis major, pectoralis minor or leg percentage, nor on prevalence of wooden breast (Table 6). A linear interaction was observed on prevalence of white striping (*P* = 0.02; Table 6). Prevalence of white striping increased linearly with an increasing breeder dietary energy-to-protein ratio in broilers from SGC breeders (β = 1.8% per % AME_n_), whereas it decreased linearly in broilers from EGC breeders (β = -1.5% per % AME_n_).

**Table 6:** Carcass yields and prevalence of breast myopathies of broilers at slaughter age (32 d of age) obtained from broiler breeders at 2 different ages (28 and 36 wk of age), which were fed to reach one of two targeted growth curves (SGC = standard growth curve or EGC = elevated growth curve [+15%]) and 4 diets, differing in energy-to-protein ratio (96, 100, 104, or 108% AME_n_), from hatch onwards.

Item		BW^1^ (g)	Carcass yield (% of BW)	Pectoralis major (% carcass)	Pectoralis minor (% carcass)	Thighs+drums (% carcass)	Wings (% carcass)	Wooden breast (%)^2^	White striping (%)^3^
Growth curve (n = 64)									
SGC		2,090	64.0	26.1	4.4	28.5	8.8	24.5	22.9
EGC		2,097	64.5	26.2	4.4	28.4	8.9	21.1	28.9
SEM		13	0.2	0.2	0.1	0.1	0.1	3.9	4.3
Diet (n = 32)									
96% AME_n_		2,082	64.2	26.1	4.4	28.4	8.9	18.8	25.0
100% AME_n_		2,095	64.3	26.2	4.3	28.4	8.8	22.4	27.1
104% AME_n_		2,092	64.2	26.1	4.5	28.3	8.8	25.0	23.4
108% AME_n_		2,105	64.2	26.2	4.3	28.6	8.7	25.0	28.1
SEM		18	0.3	0.2	0.1	0.2	0.1	5.5	6.2
Treatment (n = 16)									
SGC	96% AME_n_	2,059	63.3	25.6	4.4	29.0^a^	8.9	15.6	9.4
	100% AME_n_	2,096	64.1	26.2	4.3	28.4abc	8.8	22.9	22.9
	104% AME_n_	2,098	64.1	26.2	4.6	28.2^c^	8.7	31.3	28.1
	108% AME_n_	2,107	64.4	26.4	4.3	28.3^bc^	8.6	28.1	31.3
EGC	96% AME_n_	2,106	65.1	26.6	4.4	27.9^c^	8.8	21.9	40.6
	100% AME_n_	2,094	64.6	26.1	4.4	28.3abc	8.9	21.9	31.3
	104% AME_n_	2,086	64.2	26.0	4.4	28.5abc	8.9	18.8	18.8
	108% AME_n_	2,102	64.0	26.1	4.3	28.9^ab^	8.8	21.9	25.0
	SEM	26	0.4	0.3	0.1	0.2	0.1	7.9	8.7
Hen age (n = 64)									
28 wk		2,083	64.1	26.4	4.4	28.2^b^	9.1^a^	13.5^b^	20.6
36 wk		2,104	64.3	25.9	4.4	28.7^a^	8.5^b^	32.0^a^	31.3
SEM		13	0.2	0.2	0.1	0.1	0.1	3.9	4.3
*P*-value									
Growth curve (GC)		0.70	0.11	0.67	0.47	0.76	0.36	0.55	0.34
Diet (factorial)		0.86	0.99	0.97	0.16	0.66	0.55	0.84	0.95
Diet (linear)		0.44	0.97	0.77	0.96	0.56	0.18	0.39	0.84
Diet (quadratic)		1.00	0.92	0.86	0.47	0.29	0.60	0.74	0.84
GC x Diet (factorial)		0.66	0.09	0.23	0.43	0.005	0.71	0.67	0.10
GC x Diet (linear)		0.31	0.02	0.07	0.63	<0.001	0.30	0.32	0.02
GC x Diet (quadratic)		0.44	0.55	0.31	0.88	0.30	0.67	0.54	0.29
Hen age		0.27	0.55	0.09	1.00	0.008	<0.001	0.01	0.09

a-c LSmeans within a column and factor lacking a common superscript differ (*P* ≤ 0.05).

1 Average BW of randomly selected broilers for slaughter (per pen 2 male and 2 female broilers).

2 Percentage of broilers with score 1 (part of breast muscle is hardened) or score 2 (whole breast muscle is hardened) wooden breast.

3 Percentage of broilers with score 1 (small white lines <1 mm) or score 2 (large white lines 1–2 mm) white striping.

## DISCUSSION

The objective of this study was to evaluate effects of growth curve and dietary energy-to-protein ratio of broiler breeder hens on offspring quality and performance.

### Breeder Growth Curve

In the current study, hatching eggs were selected based on average EW per treatment. Selected hatching eggs from EGC breeders were heavier than from SGC breeders, due to a higher average EW for EGC breeders (Heijmans et al., 2021). A higher EW is probably due to a higher feed allowance of EGC breeders, compared to SGC breeders, which has been discussed previously by Heijmans et al. (2021). Although eggs were heavier from EGC breeders, no difference was observed in relative EW loss during incubation between GC. Egg weight loss is mainly determined by water loss through the eggshell during incubation and is optimal between 6.5 and 14.0% (Molenaar et al., 2010b). Egg weight loss during incubation is determined by water vapor pressure differences between the egg and its surrounding, which was similar for all eggs in the current study, and eggshell characteristics, such as eggshell thickness, number of pores or membrane characteristics (Molenaar et al., 2010b). It can be speculated that these characteristics were similar for eggs from different GC, as relative EW loss did not differ between eggs from different GC. To our knowledge, no studies are available on the impact of breeder GC on eggshell characteristics.

Broiler breeders are commonly fed restricted quantities of feed to control BW development and ensure reproductive performance (Robinson et al., 1991; Bruggeman et al., 1999; Hocking et al., 2002; Sun et al., 2006). In the current study, no effect of GC was observed on fertility, hatchability or embryonic mortality for breeders at 28 and 36 wk of age. Other studies also did not observe an effect of a higher breeder GC, compared to breeder recommendations, during rearing (28–200% higher; Hocking et al., 2002; Zuidhof et al., 2007) or during production (14% higher; Hocking et al., 2002) on fertility or hatchability. Van Emous et al. (2015a) did observe a 3.5% higher fertility and a 2.3% lower embryonic mortality at 29 wk of age of a 7.5% higher GC during rearing, but these carry-over effects disappeared at a later age (33 and 37 wk of age). In contrast to our study, other studies observed a 22% lower fertility (Hocking et al., 2002) or a 2.2 to 7.9% lower hatchability (Renema et al., 2001; Hocking et al., 2002) when breeders were on a higher GC (8% higher; Renema et al., 2001) or fed ad libitum (Hocking et al., 2002) during rearing and production. The latter studies, however, were performed over 20 yr ago and might not be applicable to modern broiler breeders. The current results suggest that feed restriction level might be reduced in modern broiler breeders without negative effects on reproductive performance.

Breeder GC did not impact chick quality scores, except for navel score. Chicks from EGC breeders had a higher prevalence of navels with black buttons (>2 mm; score 2), than chicks from SGC breeders. Elevated growth curve breeders produced heavier eggs with larger yolk, which resulted in relative and absolute larger RY at hatch. Embryos might have had more difficulties to insert the larger remaining yolk properly into the body, leading to a poorer navel closure, as observed by Molenaar et al. (2010a). A poorer navel closure is in indicator for a lower chick quality and might result in a lower posthatch performance and a higher mortality (Fasenko and O'Dea, 2008). However, this was not observed in the current study. Chicks from EGC breeders even had a better posthatch performance than chicks from SGC breeders in the current study, suggesting that other factors outbalanced the potential negative effect of navel closure on posthatch performance.

A 1.9 g heavier hatchling from EGC breeders compared to SGC breeders seems consequential to a 2.4 g larger hatching egg (Ulmer-Franco et al., 2010; Nangsuay et al., 2011; Iqbal et al., 2017). A heavier day-old chick, more specifically a heavier YFBM, is an indicator for better chick quality (reviewed by Narinç and Aydemir, 2021) and a predictor for slaughter weight (Willemsen et al., 2008). In the current study, chicks from EGC breeders maintained a higher BW up to slaughter age compared to chicks from SGC breeders, due to an 1.0 g/d higher ADG and 1.5 g/d higher ADFI. Bowling et al. (2018) also observed a higher growth, leading to a higher slaughter weight, of offspring from heavier breeders (+15% BW compared to standard), although exact growth and slaughter weight numbers are not reported in this study. In other studies, a 2.5 to 22.5% higher BW of breeders during the rearing and laying phase had no effect on hatchling weight or BW gain of the offspring (Afrouziyeh et al., 2021; Zukiwsky et al., 2021a). In these studies, however, the authors also did not observe an effect of breeder BW on EW (Afrouziyeh et al., 2021; Zukiwsky et al., 2021b). Discrepancy between these studies and the current study might be due to feeding frequency. In the studies of Afrouziyeh et al. (2021) and Zukiwsky et al. (2021a,b) breeders were fed continuously during the day with a precision feeding system, whereas in the current study breeders were fed once a day. It has been shown that continuous feeding, compared to once a day feeding, can induce metabolic changes (Van der Klein et al., 2018; Zuidhof, 2018). In turn, it has been proposed that metabolic status plays an important role in reproduction (Bédécarrats et al., 2016; Van der Klein et al., 2020), although mechanisms are not fully elucidated yet (Bédécarrats et al., 2016; Van der Klein et al., 2020).

### Breeder Dietary Energy-to-Protein Ratio

In order to achieve a similar BW, feed allocation decreased with an increasing dietary energy-to-protein ratio (Heijmans et al., 2021). With an increasing dietary energy-to-protein ratio CP intake decreased up to 14.5% and energy intake increased up to 2.3% during rearing and production (Heijmans et al., 2021). Increased breeder dietary energy-to-protein ratio decreased size of selected hatching eggs. This is most probably due to a decreasing total CP intake when dietary energy-to-protein ratio increases (Heijmans et al., 2021). No effect of dietary energy-to-protein ratio was observed on EW loss. As discussed before, this suggests eggshell characteristics, such as eggshell thickness, number of pores or membrane characteristics (Molenaar et al., 2010b) are not affected by dietary energy-to-protein ratio.

No effect of dietary energy-to-protein ratio was observed on fertility. This is in line with other studies, where authors also did not find an effect of 1.0 to 5.4% reduction in dietary CP intake during rearing (Hocking et al., 2002; Van Emous et al., 2015a, b), or 9.6 to 17.5% reduction in dietary CP intake during production (Mohiti-Asli et al., 2012; Van Emous et al., 2018), or 1.0% higher or 2.0% lower dietary energy intake during production (Van Emous et al., 2015b) on fertility. Ekmay et al. (2013) observed an effect of specifically lysine and isoleucine intake on fertility. An oversupply of either of these amino acids resulted in a decreased fertility, probably due to an increase in pH around the sperm storage tubules of the breeder hen (Ekmay et al., 2013). Lesuisse et al. (2017) observed a 14.5% lower fertility when dietary CP intake was severely reduced with 22.8% during rearing and production. These results suggest that dietary energy-to-protein ratio does not affect fertility, as long as diets have a balanced amino acid profile and CP intake is not that severely reduced (maximal 17.5%) compared to breeder recommendations.

Different breeder dietary energy-to-protein ratios resulted in a similar hatchability of fertile eggs. Several other studies also did not observe an effect of breeder dietary CP intake during rearing (Hocking et al., 2002; Van Emous et al., 2015a, b), during production (Mohiti-Asli et al., 2012; Van Emous et al., 2018), during rearing and production (Lesuisse et al., 2017) or breeder dietary energy level during production (Van Emous et al., 2015b) on hatchability of fertile eggs. Van Emous et al. (2015b) observed a 1.1% higher hatchability in the first laying phase (wk 22–45) when breeders had a 3.7% lower dietary CP intake during rearing compared to high dietary CP intake. In that same study, they also observed a 1.5% higher hatchability for breeders with an 8 to 10% lower CP intake during the second laying phase (wk 45–60). Although hatchability did not differ in the current study, very early embryonic mortality (E0-E3) decreased with an increasing breeder dietary energy-to-protein ratio. These results support the observations from Van Emous et al. (2015b), indicating a reduction in breeder CP intake might be beneficial for hatchability, due to a lower early embryonic mortality. A lower CP intake may have resulted in a lower albumen pH (Silversides and Budgell, 2004). In turn, a lower albumen pH has been related to an improved hatchability (Walsh et al., 1995; Reijrink et al., 2008). To protect the embryo from a suboptimal albumen pH, an effective barrier is formed between the ectodermal and endodermal epithelia of the embryo (Gillespie and McHanwell, 1987). Maintenance of this barrier might cause a depletion of energy reserves, particularly glucose, of the embryo (Walsh et al., 1995). During the first days of incubation an embryo mainly uses glucose as energy source (Moran, 2007). It can therefore be speculated that embryos, originating from breeders fed with a higher dietary energy-to-protein ratio, have a higher availability of glucose as they need less energy for maintenance of the barrier, due to a lower albumen pH. The higher availability of glucose for these embryos might have led to a higher survivability.

An increase in breeder dietary energy-to-protein ratio, and thus a decrease in dietary CP intake, resulted in a lower hatchling weight as a result of a lower hatching egg weight. This was also observed by Lesuisse et al. (2017). Breeders with a 22.8% lower CP intake during rearing and production produced eggs and hatchlings with a lower weight (Lesuisse et al., 2017). Van Emous et al. (2015a,b, 2018) did not find an effect of breeder dietary energy-to-protein ratio on EW nor on hatchling weight. In these studies, however, dietary energy-to-protein ratio was altered during either the rearing phase (Van Emous et al., 2015a,b) or the production phase (Van Emous et al., 2015b, 2018) alone and not in both phases, like the current study. Possibly, a lower breeder dietary energy-to-protein ratio can be beneficial for hatchling weight, but only when a lower breeder dietary energy-to-protein ratio is fed during both rearing and production.

Breeder dietary energy-to-protein ratio affected prevalence of chicks with a poorer navel closure. Prevalence of chicks with poorer navel closure (navel score 1) increased with increasing dietary energy-to-protein ratio in chicks from SGC breeders, whereas it decreased in chicks from EGC breeders. As discussed before, a larger RY might lead to a poorer navel closure (Molenaar et al., 2010a). However, RY size did not differ between treatments. It remains unclear why this interaction occurred.

Willemsen et al. (2008) observed a weak correlation (r = 0.3) between hatchling weight and market weight. In the current study, however, hatchlings were heaviest from the 96% AME_n_ breeders, compared to the other AME_n_ levels, whereas they had the lowest BW at market age. At hatch, BW decreased linearly with an increasing breeder dietary energy-to-protein ratio, whereas at market age, BW increased linearly with increasing breeder dietary energy-to-protein ratio. Broilers originating from breeders fed a higher dietary energy-to-protein ratio had a higher growth, a higher feed intake and were more efficient, than broilers originating from breeders fed a lower dietary energy-to-protein ratio. Several other studies also observed an effect of breeder dietary energy-to-protein ratio on progeny performance (Spratt and Leeson, 1987; Peebles et al., 2002; Moraes et al., 2014, 2019; Lesuisse et al., 2017, 2018). These results indicate that the maternal diet influences offspring performance, which is often referred to as transgenerational epigenetic programming (Berghof et al., 2013), where the phenotype of the offspring is matched to the maternal environment. Phenotypic changes in offspring can be induced by a modification in gene expression in specific tissues (Rao et al., 2009). Breeders that were fed a higher energy-to-protein ratio had a higher feed restriction and lower CP intake (Heijmans et al., 2021). We speculate that broilers originating from breeders with a high energy-to-protein ratio were programmed for an environment poor in CP and use dietary CP more efficiently, as this nutrient was poorly available in the maternal environment. This has led to a lower FCR and a higher growth for these broilers. In line with this hypothesis, Lesuisse et al. (2018) observed an enhanced nitrogen retention in broilers originating from breeders fed a low CP diet, compared to a high CP diet. Nitrogen is mainly retained as breast muscle tissue in broilers. Long term breast muscle growth is regulated by myogenic precursor cells, satellite cells (Halevy et al., 2000; Sklan et al., 2003; Halevy, 2020). It has been observed that satellite cell activity depends on expression of specific genes (Halevy et al., 2004) and can be altered by a change in prenatal or early posthatch environment (Halevy, 2020). It is speculated that epigenetic effects have been triggered in the current study causing an enhanced nitrogen retention by upregulation of satellite cell activity.

An increase in breeder dietary energy-to-protein ratio resulted in a higher carcass yield in offspring from SGC breeders. Moraes et al. (2019) also observed a higher carcass yield for offspring from breeders fed a higher dietary energy-to-protein ratio during rearing. As speculated before, a low CP availability in breeders, might have resulted in epigenetic changes in satellite cell activity and leading to a higher muscle growth. This might have happened in offspring from SGC breeders. However, within offspring from EGC breeders, an increase in breeder dietary energy-to-protein ratio resulted in a lower carcass yield. It remains unclear why this interaction occurred. A higher breast muscle growth has been associated with a higher occurrence of myopathies (Velleman, 2015). In line with a higher carcass yield, prevalence of white striping increased with an increasing dietary energy-to-protein ratio in broilers from SGC breeders and decreased in broilers from EGC breeders.

## CONCLUSIONS

It can be concluded that an elevated growth curve of broiler breeders during both rearing and production had no effect on fertility or hatchability, but was beneficial for hatchling weight and offspring growth up to market age. Increasing breeder dietary energy-to-protein ratio led to a significantly lower very early embryonic mortality, but had minor effects on chick quality parameters. Increasing breeder dietary energy-to-protein ratio enhanced feed intake and growth and lowered FCRc. This might be due to transgenerational epigenetic effects and an altered CP efficiency.

## Appendix Supplementary materials

Image, application 1
