# Supplementary material for: Impact of growth curve and dietary energy-to-protein ratio of broiler breeders on offspring quality and performance
Source: Poult Sci. 2022 Jul 26;101(11):102071. doi: 10.1016/j.psj.2022.102071 (PMC9489505; doi:10.1016/j.psj.2022.102071)
Supplement: Supplementary file 1 [file mmc1.docx]

# SUPPLEMENTARY INFORMATION

**Table S1**. Weekly average daily gain (ADG; g/d), average daily feed intake (ADFI; g/d) and feed conversion ratios (FCR; kg of feed/kg of BW gain) of broilers obtained from broiler breeders at 2 different ages (28 and 36 wk of age), which were fed to reach one of two targeted growth curves (SGC = standard growth curve or EGC = elevated growth curve (+15%)) and 4 diets, differing in energy-to-protein ratio (96, 100, 104, or 108% AME_n_), from hatch onwards

|  |  |  | Period^1^ | | | | | | | | | | | | | | | | | | | | | | | | |
| --- | --- | --- | --- | --- | --- | --- | --- | --- | --- | --- | --- | --- | --- | --- | --- | --- | --- | --- | --- | --- | --- | --- | --- | --- | --- | --- | --- |
|  |  |  | d 0-7 | | | d 7-14 | | | d 14-21 | | | | | | d 21-28 | | | | | | d 28-32 | | | | | |  |
| Item | |  | ADG | ADFI | FCR | ADG | ADFI | FCR | ADG | | ADFI | | FCR | | ADG | | ADFI | | FCR | | ADG | | ADFI | | FCR | |  |
| Growth curve | | |  | | | | | |  |  | |  | |  | |  | |  | |  | |  | |  | |  |  |
|  | SGC |  | 17.0^b^ | 19.1^b^ | 1.13 | 41.5^b^ | 55.3^b^ | 1.34 | 68.2 | | 97.1 | | 1.42 | | 97.8 | | 140.0 | | 1.43 | | 113.7 | | 171.2 | | 1.51 | |  |
|  | EGC |  | 17.6^a^ | 19.7^a^ | 1.12 | 42.6^a^ | 56.7^a^ | 1.33 | 69.3 | | 98.5 | | 1.42 | | 99.0 | | 141.8 | | 1.43 | | 113.8 | | 173.4 | | 1.53 | |  |
|  | SEM |  | 0.1 | 0.1 | 0.01 | 0.2 | 0.2 | 0.01 | 0.4 | | 0.4 | | 0.01 | | 0.6 | | 0.7 | | 0.01 | | 1.1 | | 1.1 | | 0.01 | |  |
| Diet | | |  | | | | | |  |  | |  | |  | |  | |  | |  | |  | |  | |  |  |
|  | 96% AME_n_ | | 17.5 | 19.6^a^ | 1.12 | 41.4^b^ | 55.4^b^ | 1.34 | 67.4 | | 96.5 | | 1.43 | | 97.0 | | 138.6 | | 1.43 | | 110.6^b^ | | 169.6 | | 1.54 | |  |
|  | 100% AME_n_ | | 17.2 | 19.2^b^ | 1.12 | 41.7^b^ | 55.8^b^ | 1.34 | 69.2 | | 97.8 | | 1.41 | | 98.4 | | 140.8 | | 1.43 | | 115.6^a^ | | 173.9 | | 1.51 | |  |
|  | 104% AME_n_ | | 17.3 | 19.5^ab^ | 1.12 | 43.0^a^ | 57.0^a^ | 1.33 | 69.6 | | 98.8 | | 1.42 | | 98.9 | | 142.2 | | 1.44 | | 112.5^ab^ | | 172.1 | | 1.53 | |  |
|  | 108% AME_n_ | | 17.2 | 19.3^b^ | 1.12 | 41.9^b^ | 55.9^b^ | 1.33 | 68.8 | | 98.2 | | 1.42 | | 99.3 | | 142.0 | | 1.43 | | 116.1^a^ | | 173.7 | | 1.50 | |  |
|  | SEM |  | 0.1 | 0.1 | 0.01 | 0.3 | 0.3 | 0.01 | 0.6 | | 0.6 | | 0.01 | | 0.9 | | 1.0 | | 0.01 | | 1.6 | | 1.6 | | 0.01 | |  |
| Treatment | | |  | | | | | |  |  | |  | |  | |  | |  | |  | |  | |  | |  |  |
|  | SGC | 96% AME_n_ | 17.0 | 19.2 | 1.13 | 40.5 | 54.4 | 1.34 | 66.4 | | 95.0^c^ | | 1.43 | | 95.4 | | 136.4 | | 1.43 | | 111.5 | | 169.4 | | 1.52 | |  |
|  |  | 100% AME_n_ | 16.8 | 18.9 | 1.13 | 40.9 | 54.8 | 1.34 | 67.6 | | 96.0^bc^ | | 1.42 | | 97.5 | | 139.4 | | 1.43 | | 114.8 | | 172.8 | | 1.51 | |  |
|  |  | 104% AME_n_ | 17.0 | 19.0 | 1.12 | 42.8 | 56.6 | 1.33 | 70.1 | | 99.3^a^ | | 1.41 | | 99.3 | | 142.4 | | 1.44 | | 111.9 | | 169.7 | | 1.52 | |  |
|  |  | 108% AME_n_ | 17.1 | 19.2 | 1.12 | 41.7 | 55.5 | 1.33 | 68.7 | | 98.1^ab^ | | 1.43 | | 98.9 | | 141.7 | | 1.43 | | 116.4 | | 173.0 | | 1.49 | |  |
|  | EGC | 96% AME_n_ | 17.9 | 20.1 | 1.12 | 42.3 | 56.5 | 1.34 | 68.4 | | 97.9^ab^ | | 1.43 | | 98.5 | | 140.8 | | 1.43 | | 109.6 | | 169.8 | | 1.55 | |  |
|  |  | 100% AME_n_ | 17.5 | 19.5 | 1.12 | 42.4 | 56.8 | 1.34 | 70.7 | | 99.7^a^ | | 1.41 | | 99.3 | | 142.1 | | 1.43 | | 116.4 | | 175.0 | | 1.50 | |  |
|  |  | 104% AME_n_ | 17.6 | 19.8 | 1.12 | 43.3 | 57.3 | 1.32 | 69.1 | | 98.3^ab^ | | 1.42 | | 98.5 | | 142.1 | | 1.44 | | 113.1 | | 174.6 | | 1.55 | |  |
|  |  | 108% AME_n_ | 17.3 | 19.4 | 1.12 | 42.2 | 56.3 | 1.33 | 69.0 | | 98.2^ab^ | | 1.42 | | 99.7 | | 142.2 | | 1.43 | | 115.9 | | 174.3 | | 1.51 | |  |
|  |  | SEM | 0.1 | 0.2 | 0.01 | 0.4 | 0.5 | 0.01 | 0.8 | | 0.9 | | 0.01 | | 1.2 | | 1.5 | | 0.01 | | 2.2 | | 2.2 | | 0.02 | |  |
| Hen age | | |  | | | | | |  |  | |  | |  | |  | |  | |  | |  | |  | |  |  |
|  | 28 weeks | | 17.0^b^ | 18.4^b^ | 1.08^b^ | 41.2^b^ | 55.9 | 1.36^a^ | 66.8^b^ | | 87.4^b^ | | 1.31^b^ | | 97.4^b^ | | 140.5 | | 1.44^a^ | | 114.1 | | 172.3 | | 1.51 | |  |
|  | 36 weeks | | 17.6^a^ | 20.4^a^ | 1.16^a^ | 42.8^a^ | 56.2 | 1.31^b^ | 70.7^a^ | | 108.2^a^ | | 1.53^a^ | | 99.3^a^ | | 141.3 | | 1.42^b^ | | 113.4 | | 172.4 | | 1.52 | |  |
|  | SEM | | 0.1 | 0.1 | 0.01 | 0.2 | 0.2 | 0.01 | 0.4 | | 0.4 | | 0.01 | | 0.6 | | 0.7 | | 0.01 | | 1.1 | | 1.1 | | 0.01 | |  |
| P-value | |  |  |  |  |  |  |  |  | |  | |  | |  | |  | |  | |  | |  | |  | |  |
|  | Growth curve (GC) | | <0.001 | <0.001 | 0.23 | <0.001 | <0.001 | 0.77 | 0.07 | | 0.03 | | 0.69 | | 0.15 | | 0.09 | | 0.95 | | 0.96 | | 0.17 | | 0.23 | |  |
|  | Diet (factorial) | | 0.14 | 0.03 | 0.99 | 0.001 | 0.02 | 0.17 | 0.07 | | 0.07 | | 0.44 | | 0.25 | | 0.08 | | 0.68 | | 0.05 | | 0.20 | | 0.15 | |  |
|  | Diet (linear) | | 0.17 | 0.11 | 0.87 | 0.03 | 0.11 | 0.15 | 0.09 | | 0.04 | | 0.79 | | 0.06 | | 0.02 | | 0.76 | | 0.07 | | 0.14 | | 0.16 | |  |
|  | Diet (quadratic) | | 0.28 | 0.18 | 0.83 | 0.03 | 0.05 | 0.41 | 0.04 | | 0.11 | | 0.12 | | 0.54 | | 0.25 | | 0.47 | | 0.66 | | 0.38 | | 0.84 | |  |
|  | GC x Diet (factorial) | | 0.09 | 0.13 | 0.70 | 0.35 | 0.26 | 0.99 | 0.08 | | 0.03 | | 0.87 | | 0.46 | | 0.39 | | 0.84 | | 0.86 | | 0.78 | | 0.81 | |  |
|  | GC x Diet (linear) | | 0.02 | 0.06 | 0.38 | 0.10 | 0.09 | 0.72 | 0.09 | | 0.03 | | 0.98 | | 0.23 | | 0.12 | | 0.79 | | 0.82 | | 0.71 | | 0.98 | |  |
|  | GC x Diet (quadratic) | | 0.62 | 0.53 | 0.85 | 0.81 | 0.87 | 0.96 | 0.91 | | 0.92 | | 0.87 | | 0.42 | | 0.57 | | 0.51 | | 0.43 | | 0.41 | | 0.60 | |  |
|  | Hen age | | <0.001 | <0.001 | <0.001 | <0.001 | 0.34 | <0.001 | <0.001 | | <0.001 | | <0.001 | | 0.03 | | 0.45 | | <0.001 | | 0.66 | | 0.93 | | 0.38 | |  |

^a-c^ LSmeans within a column and factor lacking a common superscript differ (P≤0.05).

^1^At 14 d of age, 2 adjacent pens from the same treatment were merged. n = 16 per treatment for d 0-7 and d 7-14, and n = 8 per treatment for d 14-21, d 21-28, and d 28-32.
